# Food Insecurity and Loneliness in the Former Soviet Countries

**DOI:** 10.3389/ijph.2025.1608397

**Published:** 2025-10-17

**Authors:** Andrew Stickley, Naoki Kondo, Tomiki Sumiyoshi, Mall Leinsalu, Vladislav Ruchkin, Roman Koposov, Yosuke Inoue, Martin McKee

**Affiliations:** ^1^ Stockholm Centre for Health and Social Change (SCOHOST), Sodertorn University, Huddinge, Sweden; ^2^ Department of Social Epidemiology, Graduate School of Medicine and School of Public Health, Kyoto University, Kyoto, Japan; ^3^ Department of Preventive Intervention for Psychiatric Disorders, National Institute of Mental Health, National Centre of Neurology and Psychiatry, Kodaira, Tokyo, Japan; ^4^ Department of Epidemiology and Biostatistics, National Institute for Health Development, Tallinn, Estonia; ^5^ Division of Neuroscience, Department of Child and Adolescent Psychiatry, Uppsala University, Uppsala, Sweden; ^6^ Child Study Center, Yale University School of Medicine, New Haven, CT, United States; ^7^ Sala Forensic Psychiatric Clinic, Sala, Sweden; ^8^ Regional Center for Child and Youth Mental Health and Child Welfare, Faculty of Health Sciences, UiT The Arctic University of Norway, Tromsø, Norway; ^9^ Department of Epidemiology and Modern Technologies of Vaccination, I.M. Sechenov First Moscow State Medical University, Moscow, Russia; ^10^ Independent Researcher, London, United Kingdom; ^11^ Department of Health Services Research and Policy, London School of Hygiene and Tropical Medicine, London, United Kingdom

**Keywords:** food insecure, lonely, Russia, Ukraine, cross-sectional study

## Abstract

**Objectives:**

To examine the association between food insecurity (FI) and loneliness in countries of the former Soviet Union (FSU).

**Methods:**

Data were analysed from 15,568 adults in Armenia, Azerbaijan, Belarus, Georgia, Kazakhstan, Kyrgyzstan, Moldova, Russia, and Ukraine, collected in the Health in Times of Transition (HITT) survey in 2010–2011. Information was obtained on both FI and loneliness with single-item measures. Logistic regression was used to examine associations.

**Results:**

In a fully adjusted analysis, moderate (OR: 1.35, 95% CI: 1.16–1.57) and severe FI (OR: 1.94, 95% CI: 1.58–2.38) were associated with significantly higher odds of loneliness in the pooled sample. In sex- and age-stratified analyses, severe FI was associated with loneliness in all population subgroups, with odds ratios ranging from 1.69 to 1.99. However, moderate FI was linked to loneliness in only three of the five subgroups. In further analyses, FI was associated with loneliness in six of the nine countries.

**Conclusion:**

FI is associated with loneliness in FSU countries. Efforts to increase food security in these countries may be important for improving physical and psychological well-being among adults in the general population.

## Introduction

Food insecurity (FI), which has been defined as “a household-level economic and social condition of limited or uncertain access to adequate food” [1], is widespread in countries across the world. In 2023, it was estimated that 2.33 billion people, i.e., 28.9% of the global population, were either moderately or severely food insecure [2]. This high prevalence of FI poses a significant public health challenge, given that research has linked FI to worse physical and mental health. Specifically, FI has been associated with poor self-reported health and functional disability [3], cardiometabolic disorders [4], and also with chronic pain [5] and common mental disorders such as anxiety and depression [6]. Importantly, FI has also been linked to an increased risk of premature mortality [7], with a recent study from Canada showing that among adults who died prematurely, those who were severely food insecure died on average 9 years earlier than the food-secure [8]. Various mechanisms may underlie the FI-poorer health association, including poorer diets [9] and high levels of FI-related stress [10].

More recently, studies have shown that FI is associated with other detrimental outcomes, such as poorer quality of life [11, 12] and low life satisfaction [12]. In particular, several studies have reported that there may be an association between FI and loneliness, i.e., the distressing experience that occurs when a person perceives there are deficiencies in his/her network of social relations [13]. A retrospective cohort study among a nationally representative sample of U.S. adults found that FI was associated with an increased likelihood of reporting subsequent loneliness [14]. Similarly, a large-scale study undertaken among U.S. college students aged 18–34 showed that FI was associated with 33% higher odds of loneliness [15]. Smaller-scale studies among specific population subgroups in the U.S., such as older adults living in a subsidised housing community [16] and attending healthcare clinics [17], have also linked loneliness and FI, as did a study that was focused on adults aged 18 and above living in selected Chicago community areas [18]. This association has been reported elsewhere, as a study among adults aged 50 and above in Ghana also found FI was significantly related to loneliness [19], while research among adults aged 40 and above in rural South Africa confirmed a cross-sectional association between FI and loneliness but also showed that, as FI increased over time, so did loneliness [20]. Although recent data from the United States show that there has been a growth in the number of people eating alone in the first two decades of this century, with one-quarter of adults now eating all of their daily meals alone [21], many people across the world continue to eat meals such as lunch and dinner in the company of others [21]. It is thus possible that for such people, food restrictions might affect their ability to bond with others over food and increase the possibility of experiencing loneliness [22].

The current study will examine the association between FI and loneliness in nine countries of the former Soviet Union (FSU). Given their recent histories, these countries may provide a unique context to examine the FI-loneliness association. Specifically, the economic shock that followed the collapse of the Soviet Union was accompanied by a sharp growth in poverty, declining living standards, falling farm production, and a decrease in *per capita* food consumption levels [23]. This was reflected in a high proportion of the population being undernourished in the early post-Soviet period, with figures as high as 56.5% in Georgia and over 20% in Armenia and Azerbaijan in 1990–1992 [24]. However, this situation began to reverse from 2000 when all of these countries experienced economic growth, with wages increasing greatly from the mid-2000s onwards, crucially at a faster rate than food and retail prices [25]. This was accompanied by increased access to food and higher consumption levels, with a sharp fall occurring in undernourishment levels by 2009–2011 (with the prevalence in Georgia falling to 9% and to <5% in Azerbaijan) [24].

However, at the time our study data were collected, in 2010–2011, amid growing international research interest in loneliness and its implications for wellbeing [26], the effects of the social, economic, and political changes that had occurred following the collapse of communism were still evident in our study countries. These changes, such as protracted deindustrialisation [27], which had given rise to a variety of adverse effects [28] in both the economic and social spheres [29], might have been important for outcomes such as FI and loneliness. Unemployment was prevalent, ranging from 5.6% in Azerbaijan to 27.2% in Georgia in 2010 [30]. This had its concomitant in economic hardship, as seen in poverty rates of over 20% in Moldova and Kyrgyzstan [31, 32] and over 30% in Armenia [33]. Social dislocation was also reflected in high divorce rates in Belarus, Russia, and Ukraine, and a rising divorce rate in Georgia [34]. In addition, out-migration for work was also occurring in some of these countries. For example, one estimate put the share of labour migrants among the economically active population at between 13.1% and 28.6% in Kyrgyzstan in 2010 [35]. Importantly, in 2014–2016 (the first years where country-wide estimates were available), the prevalence of moderate or severe FI in the countries in our study for which data were available ranged from 5.9% (Azerbaijan) to 31.8% (Georgia) [2], with some research suggesting that those living in poverty were especially vulnerable to FI, together with groups such as the working poor and unemployed/non-working persons [36]. Moreover, other research has shown that loneliness is common in many FSU countries [37] and is associated with adverse outcomes [38], including poorer physical and mental health [37, 39].

Given this, our first aim is therefore to determine whether FI is associated with loneliness in the FSU countries. In addition, as other research has reported that the prevalence of FI differs between men and women [2], and found that the strength of the FI-loneliness association may vary by both sex and age [19], our second aim is to explore any sex and/or age differences in this association. Finally, we will also examine if the association between FI and loneliness varies across the individual study countries.

## Methods

### Study Participants

The data came from the Health in Times of Transition (HITT) survey. This cross-country survey, which was undertaken in 2010–2011, was a follow-up to the 2001 Living Conditions, Lifestyles and Health (LLH) study [40, 41]. The HITT survey included the same eight countries that were in the LLH survey–Armenia, Belarus, Georgia, Kazakhstan, Kyrgyzstan, Moldova, Russia, and Ukraine, with the addition of Azerbaijan. The choice of study countries was guided by a variety of factors, including the availability of research collaborators and the feasibility of undertaking nationwide research. Multistage random sampling was performed, with stratification by region and urban-rural settlement type, to obtain nationally representative samples. Households were selected using random route procedures within the primary sampling units (PSUs–approximately 100–200 per country). When a household was selected, it was visited up to three times (on different days and times). If no response was obtained after the third visit, the next household on the route was selected. One individual aged ≥18 was randomly selected from within each household (using the nearest approaching or most recent past birthday) to participate in the survey. Face-to-face interviews were conducted in the respondents’ homes using a standard questionnaire that collected information on a range of variables, including demographic and household characteristics, health and healthcare utilisation, diet, tobacco and alcohol consumption, social capital, and political attitudes. The questionnaire was translated from English into Russian and each of the other country languages using standard translation techniques (forward-backward translation and expert review). Respondents could use their own country language or respond in Russian, except in Russia and Belarus, where only Russian was used. There were several exclusion criteria, i.e., being in the military, institutionalised, hospitalised, incarcerated, or homeless. Individuals were also excluded if they were intoxicated when the survey was administered. In six countries, the sample size was 1,800 respondents. However, larger samples were collected in Russia (3,000 persons) and Ukraine (2,000 persons) to reflect their larger and more regionally diverse populations. More people were also surveyed in Georgia (2,200 persons) due to a 400-person booster survey undertaken towards the end of 2010 to ensure the sample’s greater representativeness. Across the nine countries, the survey response rates ranged from 47% (in Kazakhstan) to 83% (in Georgia) [42]. Permission for the survey was obtained from the London School of Hygiene and Tropical Medicine ethics committee. Ethical permission was also obtained from our research collaborators in the study countries, i.e., the GORBI Foundation (Armenia, Azerbaijan, Georgia), the Centre for Social and Political Research, Belarusian State University (Belarus), the Centre for Study of Public Opinion (CIOM) (Kazakhstan also responsible for Kyrgyzstan), the Independent Sociological and Information Service “Opinia” (Moldova), the Open Health Institute (Russia), and the East-Ukrainian Foundation for Social Research (Ukraine) [43]. The survey was conducted in accordance with the 1964 Helsinki Declaration and subsequent amendments, with all respondents providing written informed consent before their participation.

### Food Insecurity and Loneliness

Respondents were asked about their household’s ability to obtain various goods and services. To assess FI, respondents were asked, “How often in the past 12 months did your household have to limit…Basic foods?” There were six response options: ‘constantly’, ‘sometimes’, ‘never’, ‘do not use it’, ‘do not know’, and ‘refuse to answer’. Responses were subsequently categorised as never–no FI, sometimes–moderate FI, constantly–severe FI.

Using past studies as a guide [44, 45], the subjective feeling of loneliness was assessed with a single question: “How often do you feel lonely?” (The Russian translation of this question was “Как часто Вы чувствуете себя одиноко?”) There were four response options: “never,” “rarely,” “sometimes,” and “often.” Following the lead of an earlier study that used this item when examining loneliness in these countries [37], those who responded “often” were categorised as being lonely.

### Covariates

The choice of covariates to include in the analysis was guided by previous research [14, 15] and its correspondence with the availability of variables in the HITT dataset. Information was obtained on the respondents’ sex (male, female) and age. This latter variable was subsequently categorised to represent young adulthood, i.e., age 18–34, middle age, 35–59, and older adulthood, ≥60. For educational level, respondents were categorised as having a (i) low education–incomplete secondary education or lower; (ii) mid education–completed secondary/secondary specialised education; or (iii) high education–completed/non-finished higher education. Three categories were used for marital status: (i) married/cohabiting, (ii) never married, (iii) divorced/widowed. For residential location, respondents were classified as living in either rural or urban areas. The household’s financial situation was assessed by asking, “How would you describe your household’s economic situation at present?” Answers were combined and then categorised as “good/very good,” “average,” or “bad/very bad.” Self-rated health was classified as “good/very good,” “fair,” or “poor/very poor.” Information on social support was obtained with five questions that enquired about whether the respondents had access to various types of support, such as someone who could comfort them when they were upset, would listen to them when they needed to talk, and whom they could count on to help them out in a crisis. The “yes” responses were summed to create a score between 0 and 5. These scores were then categorised as “low” social support (a score of 0–1), “medium” social support (2–3), and “high” social support (4–5). Cronbach’s alpha for the scale was 0.85. Psychological distress was assessed using a 12-item scale used in these countries previously [46, 47]. It inquires about symptoms such as feeling stressed, an inability to concentrate, insomnia, exhaustion/fatigue, etc., in the last few weeks. After removing one item on loneliness, the summed scores could range from 0 to 11, with higher scores indicating greater psychological distress. Cronbach’s alpha for the scale was 0.80.

### Statistical Analysis

We began with a descriptive analysis of FI status, where Chi-square tests were used to assess differences between the variable categories. Logistic regression was then used to examine the association between FI and loneliness in the total sample. Five models were used in the analysis. Model 1 reports the unadjusted association between FI and loneliness. In subsequent models, we added sex, age, education, marital status, household finances, residential location (Model 2), self-rated health (Model 3), social support (Model 4) and psychological distress (Model 5). Sex- and age-stratified analyses were performed using the same model-building strategy. All pooled analyses were also adjusted for the country [46, 48]. The analytic strategy described above was also used when the association between FI and loneliness was examined in each of the individual countries. Finally, to determine if the listwise deletion of missing cases had affected the results, we also undertook a sensitivity analysis where missing values were imputed. Specifically, multiple imputation by chained equation was used to generate 20 datasets, and Rubin’s rules were used to combine the estimates.

The analyses were performed with the statistical package SPSS version 24 and STATA version 18.0. Results are presented as odds ratios (OR) with 95% confidence intervals (CI). The level of statistical significance was p < 0.05.

## Results

After removing individuals who did not report how often their household had to limit food in the previous 12 months or who were missing data on any of the other variables (i.e., 2,432 cases or 13.5% of the original sample) the analytic sample consisted of 15,568 individuals with a mean (SD) age of 42.3 (17.0) years (range 18–95) with more women than men (56.5% > 43.5%). In terms of FI, 61.4% (*n* = 9,566) reported no FI, 29.9% (*n* = 4,648) reported moderate FI and 8.7% (*n* = 1,354) had severe FI. Almost 9% of the respondents were categorised as lonely. The sample characteristics by FI status are presented in Table 1, and the sample characteristics for the individual countries are presented in Supplementary Appendix 1. FI was significantly more prevalent among women, those who were older, had low education, were divorced/widowed, had bad/very bad household finances, lived in rural areas, had poor/very poor self-rated health, low social support and more psychological distress. In addition, the prevalence of FI was significantly higher among lonely than non-lonely individuals.

**TABLE 1:** Sample characteristics by food insecurity status in the countries of the former Soviet Union in 2010–2011.

Variable	Total sample	No food insecurity	Moderate food insecurity	Severe food insecurity	P-value
	*n* (%)	*n* (%)	*n* (%)	*n* (%)	
Loneliness					<0.001
No	14,185 (91.1)	9,014 (63.5)	4,098 (28.9)	1,073 (7.6)	
Yes	1,383 (8.9)	552 (39.9)	550 (39.8)	281 (20.3)	
Sex					<0.001
Men	6,770 (43.5)	4,399 (65.0)	1,841 (27.2)	530 (7.8)	
Women	8,798 (56.5)	5,167 (58.7)	2,807 (31.9)	824 (9.4)	
Age					<0.001
18–34	6,042 (38.8)	4,143 (68.6)	1,541 (25.5)	358 (5.9)	
35–59	6,760 (43.4)	4,023 (59.5)	2,138 (31.6)	599 (8.9)	
≥60	2,766 (17.8)	1,400 (50.6)	969 (35.0)	397 (14.4)	
Education					<0.001
High	4,369 (28.1)	3,051 (69.8)	1,078 (24.7)	240 (5.5)	
Mid	9,216 (59.2)	5,461 (59.3)	2,916 (31.6)	839 (9.1)	
Low	1,983 (12.7)	1,054 (53.2)	654 (33.0)	275 (13.9)	
Marital status					<0.001
Married/cohabiting	9,749 (62.6)	6,003 (61.6)	2,962 (30.4)	784 (8.0)	
Never married	3,268 (21.0)	2,257 (69.1)	790 (24.2)	221 (6.8)	
Divorced/widowed	2,551 (16.4)	1,306 (51.2)	896 (35.1)	349 (13.7)	
Household finances					<0.001
Good/very good	3,613 (23.2)	2,871 (79.5)	555 (15.4)	187 (5.2)	
Average	8,910 (57.2)	5,893 (66.1)	2,588 (29.0)	429 (4.8)	
Bad/very bad	3,045 (19.6)	802 (26.3)	1,505 (49.4)	738 (24.2)	
Location					<0.001
Urban	9,320 (59.9)	5,935 (63.7)	2,610 (28.0)	775 (8.3)	
Rural	6,248 (40.1)	3,631 (58.1)	2,038 (32.6)	579 (9.3)	
Self-rated health					<0.001
Good/very good	6,552 (42.1)	4,556 (69.5)	1,605 (24.5)	391 (6.0)	
Fair	6,269 (40.3)	3,927 (62.6)	1,908 (30.4)	434 (6.9)	
Poor/very poor	2,747 (17.6)	1,083 (39.4)	1,135 (41.3)	529 (19.3)	
Social support					<0.001
High	13,934 (89.5)	8,770 (62.9)	4,051 (29.1)	1,113 (8.0)	
Medium	1,022 (6.6)	527 (51.6)	372 (36.4)	123 (12.0)	
Low	612 (3.9)	269 (44.0)	225 (36.8)	118 (19.3)	
Psychological Distress					<0.001
M (SD)	3.02 (2.80)	2.63 (2.54)	3.44 (2.98)	4.34 (3.24)	

M, Mean; SD, Standard deviation.

When a logistic regression analysis was performed using the total pooled sample, moderate and severe FI were associated, respectively, with 2.3 and 4.9 times higher odds of loneliness in the unadjusted model (Model 1, Table 2). Adjusting the model for the covariates attenuated the strength of the association between FI and loneliness. In the fully adjusted Model 5, moderate (OR: 1.35, 95% CI: 1.16–1.57) and severe FI (OR: 1.94, 95% CI: 1.58–2.38) continued to be associated with significantly higher odds of loneliness. Regression diagnostics showed that the highest variance inflation factor (VIF) value for any of the variable categories was 2.05, indicating that multicollinearity was not an issue.

**TABLE 2:** Food insecurity and loneliness in the countries of the former Soviet Union in 2010–2011 (*n* = 15,568).

	Model 1	Model 2	Model 3	Model 4	Model 5
	OR (95% CI)	OR (95% CI)	OR (95% CI)	OR (95% CI)	OR (95% CI)
Food insecurity					
None	Ref.	Ref.	Ref.	Ref.	Ref.
Moderate	2.28 (2.00–2.59)***	1.51 (1.31–1.75)***	1.47 (1.27–1.70)***	1.41 (1.22–1.63)***	1.35 (1.16–1.57)***
Severe	4.94 (4.18–5.84)***	2.45 (2.02–2.98)***	2.27 (1.87–2.77)***	2.10 (1.72–2.57)***	1.94 (1.58–2.38)***
Sex (Female)		1.37 (1.20–1.57)***	1.30 (1.13–1.48)***	1.36 (1.19–1.57)***	1.22 (1.06–1.41)**
Age					
18–34		Ref.	Ref.	Ref.	Ref.
35–59		1.32 (1.10–1.59)**	1.14 (0.94–1.38)	1.08 (0.89–1.31)	1.03 (0.85–1.25)
≥60		2.05 (1.67–2.51)***	1.52 (1.22–1.89)***	1.40 (1.12–1.75)**	1.40 (1.12–1.75)**
Education					
High		Ref.	Ref.	Ref.	Ref.
Mid		1.16 (0.99–1.36)	1.12 (0.95–1.31)	1.08 (0.92–1.28)	1.09 (0.92–1.28)
Low		1.32 (1.08–1.62)**	1.21 (0.98–1.49)	1.09 (0.88–1.35)	1.02 (0.82–1.26)
Marital status					
Married/cohabiting		Ref.	Ref.	Ref.	Ref.
Never married		2.06 (1.68–2.53)***	2.14 (1.74–2.62)***	2.01 (1.63–2.47)***	2.02 (1.64–2.50)***
Divorced/widowed		5.37 (4.64–6.21)***	5.31 (4.58–6.15)***	4.76 (4.10–5.54)***	4.80 (4.12–5.59)***
Household finances					
Good/very good		Ref.	Ref.	Ref.	Ref.
Average		1.43 (1.18–1.74)***	1.34 (1.10–1.64)**	1.34 (1.10–1.64)**	1.31 (1.07–1.60)*
Bad/very bad		2.63 (2.11–3.27)***	2.16 (1.73–2.71)***	1.91 (1.52–2.40)***	1.65 (1.31–2.09)***
Location (Rural)		1.14 (1.00–1.30)*	1.12 (0.98–1.28)	1.10 (0.97–1.26)	1.10 (0.96–1.26)
Self-rated health					
Good/very good			Ref.	Ref.	Ref.
Fair			1.33 (1.12–1.58)**	1.40 (1.18–1.67)***	1.12 (0.93–1.34)
Poor/very poor			2.29 (1.88–2.79)***	2.40 (1.96–2.93)***	1.25 (1.01–1.56)*
Social support					
High				Ref.	Ref.
Medium				2.30 (1.90–2.80)***	2.14 (1.75–2.61)***
Low				5.12 (4.15–6.31)***	4.74 (3.82–5.89)***
Psychological distress					1.22 (1.19–1.25)***
Pseudo *R* ^*2*^ (Nagelkerke)	0.08	0.24	0.25	0.28	0.31

OR, Odds ratio; CI, Confidence interval; Ref, Reference category; *p < 0.05, **p < 0.01, ***p < 0.001; All models were adjusted for country.

In the sex- and age-stratified analyses, in the fully adjusted Model 5, severe FI was associated with significantly higher odds of loneliness across all groups with ORs ranging from 1.69 among adults aged 18–34 to 1.99 in those aged 35–59 (Table 3). In contrast, there was more variation for moderate FI, which was not significantly associated with loneliness in men or those aged 35–59, but had the strongest association with loneliness in individuals aged 18–34.

**TABLE 3:** Sex- and age-specific associations between food insecurity and loneliness in the countries of the former Soviet Union in 2010–2011.

	Model 1	Model 2	Model 3	Model 4	Model 5
	OR (95% CI)	OR (95% CI)	OR (95% CI)	OR (95% CI)	OR (95% CI)
Sex					
Men (*n* = 6,770)					
Food insecurity					
None	Ref.	Ref.	Ref.	Ref.	Ref.
Moderate	1.95 (1.54–2.47)***	1.29 (0.99–1.67)	1.25 (0.96–1.63)	1.19 (0.91–1.56)	1.15 (0.87–1.51)
Severe	4.34 (3.19–5.90)***	2.41 (1.70–3.42)***	2.31 (1.63–3.29)***	2.10 (1.47–3.01)***	1.89 (1.31–2.73)**
Women (*n* = 8,798)					
Food insecurity					
None	Ref.	Ref.	Ref.	Ref.	Ref.
Moderate	2.32 (1.98–2.71)***	1.61 (1.36–1.91)***	1.56 (1.31–1.86)***	1.51 (1.27–1.80)***	1.44 (1.20–1.72)***
Severe	4.97 (4.06–6.08)***	2.49 (1.97–3.15)***	2.28 (1.80–2.89)***	2.12 (1.66–2.70)***	1.97 (1.54–2.53)***
Age					
18–34 (*n* = 6,042)					
Food insecurity					
None	Ref.	Ref.	Ref.	Ref.	Ref.
Moderate	2.56 (1.97–3.32)***	2.02 (1.53–2.66)***	1.96 (1.48–2.59)***	1.91 (1.44–2.54)***	1.86 (1.39–2.48)***
Severe	3.12 (2.04–4.77)***	2.39 (1.52–3.75)***	2.24 (1.43–3.53)***	1.96 (1.23–3.12)**	1.69 (1.04–2.72)*
35–59 (*n* = 6,760)					
Food insecurity					
None	Ref.	Ref.	Ref.	Ref.	Ref.
Moderate	1.86 (1.51–2.29)***	1.33 (1.06–1.66)*	1.27 (1.01–1.60)*	1.18 (0.94–1.49)	1.09 (0.86–1.39)
Severe	4.54 (3.49–5.90)***	2.74 (2.03–3.70)***	2.54 (1.88–3.44)***	2.31 (1.70–3.14)***	1.99 (1.45–2.73)***
≥60 (*n* = 2,766)					
Food insecurity					
None	Ref.	Ref.	Ref.	Ref.	Ref.
Moderate	1.90 (1.52–2.37)***	1.41 (1.10–1.81)**	1.39 (1.08–1.78)*	1.37 (1.06–1.77)*	1.35 (1.04–1.75)*
Severe	3.58 (2.72–4.71)***	2.16 (1.57–2.98)***	1.98 (1.43–2.73)***	1.93 (1.38–2.71)***	1.95 (1.38–2.76)***

OR, Odds ratio; CI, Confidence interval; Ref, Reference category.

Model 1 examined the bivariate association between food insecurity and loneliness; Other models were additionally adjusted for sex, age (where appropriate) education, marital status, household financial situation, location (Model 2) self-rated health (Model 3), social support (Model 4), psychological distress (Model 5). All models were adjusted for country.

*p < 0.05, **p < 0.01, ***p < 0.001.

The results for FI from the final model (Model 5) of the individual country analyses are presented in Table 4. Severe FI was associated with loneliness in six countries and moderate FI was linked to loneliness in three countries. In Armenia, Kazakhstan, and Russia both moderate and severe FI were associated with significantly higher odds of loneliness. In contrast, in Belarus, Georgia and Moldova neither moderate nor severe FI were significantly associated with loneliness. The full results for each country across the five models are presented in online Supplementary Appendix 2.

**TABLE 4:** Association between food insecurity and loneliness in nine former Soviet Union countries in 2010–2011.

	Armenia (*n* = 1,501)	Azerbaijan (*n* = 1,623)	Belarus (*n* = 1,573)	Georgia (*n* = 2,006)	Kazakhstan (*n* = 1,616)	Kyrgyzstan (*n* = 1,719)	Moldova (*n* = 1,560)	Russia (*n* = 2,336)	Ukraine (*n* = 1,634)
	OR (95% CI)	OR (95% CI)	OR (95% CI)	OR (95% CI)	OR (95% CI)	OR (95% CI)	OR (95% CI)	OR (95% CI)	OR (95% CI)
Food insecurity									
None	Ref.	Ref.	Ref.	Ref.	Ref.	Ref.	Ref.	Ref.	Ref.
Moderate	1.67 (1.09–2.58)*	1.16 (0.61–2.21)	0.96 (0.56–1.64)	1.10 (0.71–1.70)	2.38 (1.35–4.19)**	1.27 (0.79–2.04)	1.07 (0.75–1.52)	2.00 (1.33–2.99)**	1.33 (0.84–2.12)
Severe	2.07 (1.15–3.70)*	2.50 (1.20–5.23)*	1.26 (0.51–3.09)	1.27 (0.74–2.16)	3.84 (1.48–9.98)**	2.12 (1.23–3.66)**	1.28 (0.62–2.64)	3.60 (1.90–6.82)***	2.68 (1.50–4.78)**

For each country the analysis was adjusted for sex, age, education, marital status, household financial situation, location, self-rated health, social support, psychological distress.

*p < 0.05, **p < 0.01, ***p < 0.001.

Finally, when multiple imputation was used to assess if the listwise deletion of missing cases had affected the outcome, it showed that the results were essentially the same. For instance, for the main analysis, the OR for moderate FI was 1.35 (95% CI: 1.18–1.55) and for severe FI 1.91 (95% CI: 1.59–2.31) (data not tabulated).

## Discussion

We examined the link between FI and loneliness across nine post-Soviet countries. Over one-third (38.6%) of respondents experienced moderate or severe FI. A fully adjusted logistic regression analysis showed that both moderate and severe FI were significantly associated with higher odds of loneliness in the total sample. In subgroup analyses by sex and age, severe FI was consistently linked to loneliness across all groups. Moderate FI showed more variability, being significant in fewer subgroups, though confidence intervals overlapped across analyses. FI was associated with loneliness in six countries, while there was no association in three countries.

In the total sample, both FI levels were significantly associated with loneliness, aligning with findings from a U.S. study using nationally representative data, where FI was linked to loneliness in both linear probability and logistic regression models [14]. Our observed 35%–94% increase in the odds of loneliness with FI is comparable to the findings from other studies. For example, FI was linked to 33% higher odds of loneliness among U.S. university students [15], and 2.52 times higher odds among rural South African adults aged 40 and above [20]. The increasing odds from moderate to severe FI, with non-overlapping confidence intervals, suggest a possible dose-response relationship, at least in the total population. To the best of our knowledge, no prior study has explored whether the strength of the FI-loneliness association varies by FI severity categories. However, this trend is plausible, as a previous analysis of the 2014 Gallup World Poll found a dose-response relationship between FI (mild, moderate, severe) and poorer mental health, measured by the negative and positive experiences indexes [49]. Future research should investigate whether FI severity differentially impacts loneliness.

Several mechanisms may explain the link between FI and loneliness. FI can limit participation in social activities and sharing food, potentially leading to loneliness [50]. It is also often stigmatising, causing shame and social isolation [50], which may contribute to loneliness. FI has been associated with reduced self-esteem [51], which is relevant given evidence that low self-esteem and loneliness can influence each other reciprocally, but that low self-esteem may be more strongly linked to later loneliness than the reverse [52]. Additionally, a study in Ghana among adults aged 50 and above identified mediators in the FI-loneliness pathway, including depression/anxiety, feelings of hopelessness, worthlessness, functional limitations, and pain severity [19].

In sex- and age-stratified analyses, severe FI was significantly associated with loneliness across all subgroups, with ORs ranging from 1.69 to 1.99. Moderate FI showed more variability, being linked to loneliness only in some groups–particularly women. This suggests the FI-loneliness association may be stronger in women, supported by findings from Ghana, where food-insecure women had higher loneliness coefficients than men [19]. Gendered roles may contribute to this difference. As primary caregivers, women may experience greater psychological and self-stigmatising effects when unable to feed their families [50], potentially increasing their desire to avoid others and thus loneliness. A Russian study found that women, especially if they are household heads or single parents, are more vulnerable to FI [36], while other research from Russia identified unmarried women with children as being at a higher risk of loneliness [53]. It is uncertain why there was no significant link between moderate FI and loneliness in men although it may reflect a variety of factors such as differences in food provision roles, perceptions of FI severity, or the lesser impact of moderate FI on men’s social engagement. Regarding age, research from Russia has indicated that older adults are more vulnerable to FI [36], while findings from Ghana suggest the FI-loneliness link is stronger in older populations [19]. Although FI was more prevalent among older adults in this study, the strength of the association between the FI categories and loneliness varied across age groups. The reasons for this variation are unclear but may relate to life course differences. For instance, unemployment, identified as a risk factor for FI in the study countries [36, 54], varies by age [55], and its relationship with loneliness may also differ across age groups [56]. Further research is needed to explore how FI and loneliness interact across different ages and to identify underlying factors.

The association between FI and loneliness varied across the nine countries studied, though the reasons remain unclear. For example, severe FI was low in both Belarus and Moldova (where no association was found), but also in Kazakhstan, where a strong association was observed (see Supplementary Appendix 1). Similarly, rising food prices in 2010 were noted in countries with and without a significant FI-loneliness link, such as Russia and Belarus [57]. However, recent research suggests that cultural factors, possibly relating to the social importance of food, may play a role. Specifically, Gallup data from 2022 to 2023 showed that among our study countries, those where the strongest FI-loneliness associations were observed, i.e., in Kazakhstan, Armenia, and Russia, also had the highest number of shared meals per week. In contrast, Georgia, where no association was seen, had the lowest [21]. This implies that the communal role of food may be one factor that influences the strength of the FI-loneliness relationship in post-Soviet countries. More research is now needed within these individual countries to further elucidate the association between between FI and loneliness and the factors that underlie it.

This study is the first to examine the link between FI and loneliness across all adult age groups in multiple post-Soviet countries. Despite its strengths, several limitations should be noted. First, FI was assessed using a single-item measure, which may underestimate prevalence [58], as multi-item tools tend to yield higher rates [59]. Second, it is possible that we lacked data on important variables that confounded or mediated the association between FI and loneliness. For example, previous research has shown that housing tenure (private renting) is associated with both FI [60] and loneliness [61]. Future research should aim to collect data on a broader set of variables than was used in this study that may be relevant to the FI-loneliness relationship, and also identify potential instrumental variables such as local food availability or access to food that are associated with FI but not directly with loneliness, thereby enabling a potentially more accurate estimation of the independent effect of FI. Third, given that FI and loneliness are both associated with social stigma [16], it is possible that respondents underreported these phenomena, i.e., social desirability bias may have been an issue. Fourth, although phenomena such as loneliness are increasingly being examined in multicountry studies [62], the use of a standard questionnaire translated into different country languages may have failed to capture cultural and linguistic differences in how participants interpret and respond to such experiences across the nine countries. In particular, the concept of loneliness may carry different cultural connotations, potentially affecting response accuracy. Fifth, as this study used cross-sectional data, it was impossible to establish causality or determine the directionality of the observed associations. Finally, these data were collected over a decade ago. Since that time, global events such as the COVID-19 pandemic and events specific to the region, such as the ongoing war in Ukraine, may have affected FI not only in Ukraine [63] but also possibly in some other countries in the region [2]. Thus, more research is needed to determine the prevalence and impact of FI in these countries today.

In conclusion, we have shown that FI was prevalent in FSU countries in the early 2010s and that individuals who experienced both moderate and severe FI had higher odds of loneliness. While we cannot establish the direction of causality, we can say that measures to reduce FI and loneliness would bring mutually reinforcing benefits.
