# Supplementary material for: Food Insecurity and Loneliness in the Former Soviet Countries
Source: Int J Public Health. 2025 Oct 17;70:1608397. doi: 10.3389/ijph.2025.1608397 (PMC12575212; doi:10.3389/ijph.2025.1608397)
Supplement: Supplementary file 1 [file Supplementaryfile1.docx]

**Appendix 1** Sample characteristics for each country included in the study

|  | Armenia  (1501) ^a^ | Azerbaijan  (1623) ^a^ | Belarus  (1573) ^a^ | Georgia  (2006) ^a^ | Kazakhstan  (1616) ^a^ | Kyrgyzstan  (1719) ^a^ | Moldova  (1560) ^a^ | Russia  (2336) ^a^ | Ukraine  (1634) ^a^ |
| --- | --- | --- | --- | --- | --- | --- | --- | --- | --- |
|  | *n* (%) | *n* (%) | *n* (%) | *n* (%) | *n* (%) | *n* (%) | *n* (%) | *n* (%) | *n* (%) |
| Characteristic |  |  |  |  |  |  |  |  |  |
| Food insecurity |  |  |  |  |  |  |  |  |  |
| None | 834 (55.6) | 828 (51.0) | 1210 (76.9) | 722 (36.0) | 1280 (79.2) | 909 (52.9) | 1054 (67.6) | 1747 (74.8) | 982 (60.1) |
| Moderate | 522 (34.8) | 592 (36.5) | 312 (19.8) | 950 (47.4) | 288 (17.8) | 564 (32.8) | 447 (28.7) | 490 (21.0) | 483 (29.6) |
| Severe | 145 (9.7) | 203 (12.5) | 51 (3.2) | 334 (16.7) | 48 (3.0) | 246 (14.3) | 59 (3.8) | 99 (4.2) | 169 (10.3) |
| Lonely (often) | 154 (10.3) | 66 (4.1) | 119 (7.6) | 232 (11.6) | 83 (5.1) | 131 (7.6) | 268 (17.2) | 172 (7.4) | 158 (9.7) |
| Sex |  |  |  |  |  |  |  |  |  |
| Male | 684 (45.6) | 756 (46.6) | 693 (44.1) | 738 (36.8) | 772 (47.8) | 829 (48.2) | 682 (43.7) | 938 (40.2) | 678 (41.5) |
| Female | 817 (54.4) | 867 (53.4) | 880 (55.9) | 1268 (63.2) | 844 (52.2) | 890 (51.8) | 878 (56.3) | 1398 (59.8) | 956 (58.5) |
| Age |  |  |  |  |  |  |  |  |  |
| 18-34 | 639 (42.6) | 721 (44.4) | 628 (39.9) | 581 (29,0) | 672 (41.6) | 822 (47.8) | 593 (38.0) | 800 (34.2) | 586 (35.9) |
| 35-59 | 648 (43.2) | 755 (46.5) | 646 (41.1) | 976 (48.7) | 715 (44.2) | 706 (41.1) | 669 (42.9) | 1023 (43.8) | 622 (38.1) |
| ≥ 60 | 214 (14.3) | 147 (9.1) | 299 (19.0) | 449 (22.4) | 229 (14.2) | 191 (11.1) | 298 (19.1) | 513 (22.0) | 426 (26.1) |
| Education level |  |  |  |  |  |  |  |  |  |
| High | 339 (22.6) | 392 (24.2) | 413 (26.3) | 779 (38.8) | 477 (29.5) | 408 (23.7) | 369 (23.7) | 630 (27.0) | 562 (34.4) |
| Mid | 1029 (68.6) | 1117 (68.8) | 1047 (66.6) | 1072 (53.4) | 957 (59.2) | 913 (53.1) | 762 (48.8) | 1432 (61.3) | 887 (54.3) |
| Low | 133 (8.9) | 114 (7.0) | 113 (7.2) | 155 (7.7) | 182 (11.3) | 398 (23.2) | 429 (27.5) | 274 (11.7) | 185 (11.3) |
| Marital status |  |  |  |  |  |  |  |  |  |
| Married/cohabiting | 976 (65.0) | 1078 (66.4) | 882 (56.1) | 1277 (63.7) | 1012 (62.6) | 1190 (69.2) | 973 (62.4) | 1414 (60.5) | 947 (58.0) |
| Never married | 405 (27.0) | 433 (26.7) | 382 (24.3) | 365 (18.2) | 362 (22.4) | 322 (18.7) | 284 (18.2) | 412 (17.6) | 303 (18.5) |
| Divorced/widowed | 120 (8.0) | 112 (6.9) | 309 (19.6) | 364 (18.1) | 242 (15.0) | 207 (12.0) | 303 (19.4) | 510 (21.8) | 384 (23.5) |
| Household finances |  |  |  |  |  |  |  |  |  |
| Good/very good | 436 (29.0) | 434 (26.7) | 372 (23.6) | 112 (5.6) | 511 (31.6) | 599 (34.8) | 429 (27.5) | 435 (18.6) | 285 (17.4) |
| Average | 806 (53.7) | 841 (51.8) | 1010 (64.2) | 1031 (51.4) | 996 (61.6) | 946 (55.0) | 756 (48.5) | 1548 (66.3) | 976 (59.7) |
| Bad/very bad | 259 (17.3) | 348 (21.4) | 191 (12.1) | 863 (43.0) | 109 (6.7) | 174 (10.1) | 375 (24.0) | 353 (15.1) | 373 (22.8) |
| Location |  |  |  |  |  |  |  |  |  |
| Urban | 1191 (79.3) | 887 (54.7) | 1164 (74.0) | 947 (47.2) | 906 (56.1) | 781 (45.4) | 599 (38.4) | 1691 (72.4) | 1154 (70.6) |
| Rural | 310 (20.7) | 736 (45.3) | 409 (26.0) | 1059 (52.8) | 710 (43.9) | 938 (54.6) | 961 (61.6) | 645 (27.6) | 480 (29.4) |
| Self-rated health |  |  |  |  |  |  |  |  |  |
| Good/very good | 839 (55.9) | 979 (60.3) | 574 (36.5) | 510 (25.4) | 727 (45.0) | 920 (53.5) | 599 (38.4) | 843 (36.1) | 561 (34.3) |
| Fair | 495 (33.0) | 378 (23.3) | 795 (50.5) | 784 (39.1) | 743 (46.0) | 598 (34.8) | 610 (39.1) | 1136 (48.6) | 730 (44.7) |
| Poor/very poor | 167 (11.1) | 266 (16.4) | 204 (13.0) | 712 (35.5) | 146 (9.0) | 201 (11.7) | 351 (22.5) | 357 (15.3) | 343 (21.0) |
| Social support |  |  |  |  |  |  |  |  |  |
| High | 1097 (73.1) | 1422 (87.6) | 1451 (92.2) | 1929 (96.2) | 1483 (91.8) | 1558 (90.6) | 1319 (84.6) | 2170 (92.9) | 1505 (92.1) |
| Middle | 288 (19.2) | 112 (6.9) | 82 (5.2) | 49 (2.4) | 103 (6.4) | 121 (7.0) | 105 (6.7) | 98 (4.2) | 64 (3.9) |
| Low | 116 (7.7) | 89 (5.5) | 40 (2.5) | 28 (1.4) | 30 (1.9) | 40 (2.3) | 136 (8.7) | 68 (2.9) | 65 (4.0) |
| Psychological distress ^b^ | 3.00 (2.85) | 2.23 (2.51) | 3.10 (2.73) | 3.12 (2.98) | 2.40 (2.47) | 3.82 (2.55) | 3.72 (3.01) | 2.71 (2.77) | 3.17 (2.88) |

^a^ The number in parentheses is the number of participants in each country; ^b^ The mean score is presented with its standard deviation in parentheses
