# Supplementary material for: Food Insecurity and Loneliness in the Former Soviet Countries
Source: Int J Public Health. 2025 Oct 17;70:1608397. doi: 10.3389/ijph.2025.1608397 (PMC12575212; doi:10.3389/ijph.2025.1608397)
Supplement: Supplementary file 2 [file Supplementaryfile2.docx]

**Appendix 2** Association between food insecurity and loneliness in the individual study countries

**Table 1.** Food insecurity and loneliness in Armenia (*n* = 1501)

|  |  | **Model 1** |  | **Model 2** |  | **Model 3** |  | **Model 4** | **Model 5** |
| --- | --- | --- | --- | --- | --- | --- | --- | --- | --- |
|  |  | OR (95%CI) |  | OR (95%CI) |  | OR (95%CI) |  | OR (95%CI) | OR (95%CI) |
| Food insecurity |  |  |  |  |  |  |  |  |  |
| None |  | Ref. |  | Ref. |  | Ref. |  | Ref. | Ref. |
| Moderate |  | 2.55 (1.75-3.72)*** |  | 1.87 (1.23-2.84)** |  | 1.79 (1.17-2.75)** |  | 1.70 (1.10-2.61)* | 1.67 (1.09-2.58)* |
| Severe |  | 4.26 (2.61-6.96)*** |  | 3.09 (1.79-5.33)*** |  | 2.80 (1.60-4.89)*** |  | 2.43 (1.38-4.29)** | 2.07 (1.15-3.70)* |
| Sex (Female) |  |  |  | 1.03 (0.71-1.49) |  | 1.00 (0.69-1.45) |  | 1.01 (0.69-1.47) | 0.98 (0.67-1.44) |
| Age |  |  |  |  |  |  |  |  |  |
| 18-34 |  |  |  | Ref. |  | Ref. |  | Ref. | Ref. |
| 35-59 |  |  |  | 1.09 (0.65-1.80) |  | 1.00 (0.59-1.67) |  | 0.96 (0.57-1.61) | 0.96 (0.57-1.62) |
| ≥60 |  |  |  | 1.77 (0.97-3.23) |  | 1.34 (0.71-2.53) |  | 1.19 (0.62-2.27) | 1.21 (0.63-2.32) |
| Education |  |  |  |  |  |  |  |  |  |
| High |  |  |  | Ref. |  | Ref. |  | Ref. | Ref. |
| Mid |  |  |  | 1.07 (0.66-1.73) |  | 1.02 (0.63-1.64) |  | 0.97 (0.60-1.57) | 1.06 (0.65-1.72) |
| Low |  |  |  | 1.05 (0.53-2.10) |  | 0.94 (0.46-1.90) |  | 0.93 (0.45-1.92) | 0.91 (0.44-1.89) |
| Marital status |  |  |  |  |  |  |  |  |  |
| Married/cohabiting |  |  |  | Ref. |  | Ref. |  | Ref. | Ref. |
| Never married |  |  |  | 1.52 (0.89-2.59) |  | 1.55 (0.91-2.65) |  | 1.58 (0.92-2.72) | 1.68 (0.97-2.89) |
| Divorced/widowed |  |  |  | 5.26 (3.17-8.73)*** |  | 5.14 (3.06-8.64)*** |  | 5.32 (3.14-9.04)*** | 5.15 (3.02-8.79)*** |
| Household finances |  |  |  |  |  |  |  |  |  |
| Good/very good |  |  |  | Ref. |  | Ref. |  | Ref. | Ref. |
| Average |  |  |  | 0.88 (0.55-1.39) |  | 0.82 (0.52-1.32) |  | 0.87 (0.54-1.40) | 0.89 (0.55-1.43) |
| Bad/very bad |  |  |  | 1.81 (1.07-3.06)* |  | 1.43 (0.82-2.49) |  | 1.40 (0.80-2.44) | 1.39 (0.79-2.44) |
| Location (Rural) |  |  |  | 0.99 (0.64-1.54) |  | 0.98 (0.63-1.53) |  | 1.04 (0.66-1.63) | 0.92 (0.58-1.46) |
| Self-rated health |  |  |  |  |  |  |  |  |  |
| Good/very good |  |  |  |  |  | Ref. |  | Ref. | Ref. |
| Fair |  |  |  |  |  | 0.93 (0.60-1.46) |  | 1.05 (0.66-1.65) | 0.84 (0.52-1.36) |
| Poor/very poor |  |  |  |  |  | 2.49 (1.45-4.30)** |  | 2.83 (1.62-4.93)*** | 1.50 (0.77-2.94) |
| Social support |  |  |  |  |  |  |  |  |  |
| High |  |  |  |  |  |  |  | Ref. | Ref. |
| Medium |  |  |  |  |  |  |  | 1.72 (1.11-2.68)* | 1.78 (1.14-2.78)* |
| Low |  |  |  |  |  |  |  | 2.40 (1.35-4.28)** | 2.55 (1.42-4.57)** |
| Psychological distress |  |  |  |  |  |  |  |  | 1.14 (1.06-1.23)** |
| Pseudo *R^2^* (Nagelkerke) |  | .06 |  | .16 |  | .18 |  | .20 | .21 |

OR: Odds ratio; CI: Confidence interval; Ref: Reference category; * p<.05, ** p<.01, *** p<.001.

**Table 2.**  Food insecurity and loneliness in Azerbaijan (*n* = 1623)

|  |  | **Model 1** |  | **Model 2** |  | **Model 3** |  | **Model 4** | **Model 5** |
| --- | --- | --- | --- | --- | --- | --- | --- | --- | --- |
|  |  | OR (95%CI) |  | OR (95%CI) |  | OR (95%CI) |  | OR (95%CI) | OR (95%CI) |
| Food insecurity |  |  |  |  |  |  |  |  |  |
| None |  | Ref. |  | Ref. |  | Ref. |  | Ref. | Ref. |
| Moderate |  | 1.48 (0.83-2.65) |  | 1.10 (0.60-2.04) |  | 1.10 (0.59-2.04) |  | 1.16 (0.62-2.18) | 1.16 (0.61-2.21) |
| Severe |  | 3.61 (1.93-6.78)*** |  | 2.46 (1.24-4.89)* |  | 2.36 (1.18-4.73)* |  | 2.40 (1.18-4.87)* | 2.50 (1.20-5.23)* |
| Sex (Female) |  |  |  | 1.46 (0.80-2.66) |  | 1.40 (0.76-2.57) |  | 1.41 (0.76-2.61) | 1.28 (0.68-2.42) |
| Age |  |  |  |  |  |  |  |  |  |
| 18-34 |  |  |  | Ref. |  | Ref. |  | Ref. | Ref. |
| 35-59 |  |  |  | 0.93 (0.45-1.92) |  | 0.79 (0.38-1.65) |  | 0.84 (0.40-1.76) | 0.73 (0.34-1.59) |
| ≥60 |  |  |  | 1.26 (0.49-3.20) |  | 0.83 (0.31-2.24) |  | 0.83 (0.31-2.27) | 1.14 (0.42-3.13) |
| Education |  |  |  |  |  |  |  |  |  |
| High |  |  |  | Ref. |  | Ref. |  | Ref. | Ref. |
| Mid |  |  |  | 1.16 (0.55-2.42) |  | 1.17 (0.56-2.46) |  | 1.08 (0.51-2.32) | 1.17 (0.54-2.52) |
| Low |  |  |  | 1.47 (0.51-4.23) |  | 1.47 (0.51-4.25) |  | 1.39 (0.47-4.13) | 1.14 (0.37-3.49) |
| Marital status |  |  |  |  |  |  |  |  |  |
| Married/cohabiting |  |  |  | Ref. |  | Ref. |  | Ref. | Ref. |
| Never married |  |  |  | 1.55 (0.69-3.51) |  | 1.71 (0.75-3.92) |  | 1.77 (0.76-4.12) | 1.96 (0.83-4.67) |
| Divorced/widowed |  |  |  | 5.75 (2.93-11.27)*** |  | 6.28 (3.17-12.41)*** |  | 5.12 (2.56-10.27)*** | 4.60 (2.24-9.44)*** |
| Household finances |  |  |  |  |  |  |  |  |  |
| Good/very good |  |  |  | Ref. |  | Ref. |  | Ref. | Ref. |
| Average |  |  |  | 1.85 (0.74-4.64) |  | 1.63 (0.64-4.14) |  | 1.65 (0.64-4.23) | 1.17 (0.45-3.07) |
| Bad/very bad |  |  |  | 5.38 (2.13-13.57)*** |  | 4.11 (1.59-10.64)** |  | 3.70 (1.40-9.76)** | 2.16 (0.79-5.93) |
| Location (Rural) |  |  |  | 1.02 (0.58-1.78) |  | 0.99 (0.56-1.74) |  | 0.93 (0.52-1.66) | 1.43 (0.77-2.65) |
| Self-rated health |  |  |  |  |  |  |  |  |  |
| Good/very good |  |  |  |  |  | Ref. |  | Ref. | Ref. |
| Fair |  |  |  |  |  | 1.62 (0.81-3.23) |  | 1.54 (0.76-3.12) | 1.21 (0.58-2.53) |
| Poor/very poor |  |  |  |  |  | 2.52 (1.25-5.11)* |  | 2.57 (1.26-5.24)* | 1.22 (0.56-2.67) |
| Social support |  |  |  |  |  |  |  |  |  |
| High |  |  |  |  |  |  |  | Ref. | Ref. |
| Medium |  |  |  |  |  |  |  | 2.56 (1.23-5.34)* | 2.58 (1.22-5.47)* |
| Low |  |  |  |  |  |  |  | 3.87 (1.82-8.24)*** | 3.16 (1.43-6.99)** |
| Psychological distress |  |  |  |  |  |  |  |  | 1.34 (1.21-1.48)*** |
| Pseudo *R^2^* (Nagelkerke) |  | .03 |  | .17 |  | .19 |  | .22 | .28 |

OR: Odds ratio; CI: Confidence interval; Ref: Reference category; * p<.05, ** p<.01, *** p<.001.

**Table 3.** Food insecurity and loneliness in Belarus (*n* = 1573)

|  |  | **Model 1** |  | **Model 2** |  | **Model 3** |  | **Model 4** | **Model 5** |
| --- | --- | --- | --- | --- | --- | --- | --- | --- | --- |
|  |  | OR (95%CI) |  | OR (95%CI) |  | OR (95%CI) |  | OR (95%CI) | OR (95%CI) |
| Food insecurity |  |  |  |  |  |  |  |  |  |
| None |  | Ref. |  | Ref. |  | Ref. |  | Ref. | Ref. |
| Moderate |  | 2.47 (1.64-3.73)*** |  | 1.26 (0.78-2.04) |  | 1.16 (0.71-1.90) |  | 1.15 (0.69-1.90) | 0.96 (0.56-1.64) |
| Severe |  | 4.62 (2.27-9.40)*** |  | 1.27 (0.55-2.95) |  | 1.01 (0.42-2.42) |  | 1.15 (0.48-2.78) | 1.26 (0.51-3.09) |
| Sex (Female) |  |  |  | 2.14 (1.33-3.42)** |  | 2.13 (1.32-3.43)** |  | 2.47 (1.50-4.06)*** | 2.05 (1.23-3.43)** |
| Age |  |  |  |  |  |  |  |  |  |
| 18-34 |  |  |  | Ref. |  | Ref. |  | Ref. | Ref. |
| 35-59 |  |  |  | 1.77 (0.88-3.57) |  | 1.59 (0.77-3.31) |  | 1.41 (0.68-2.93) | 1.44 (0.65-3.17) |
| ≥60 |  |  |  | 2.84 (1.35-5.98)** |  | 2.14 (0.97-4.76) |  | 1.89 (0.85-4.20) | 2.32 (0.99-5.45) |
| Education |  |  |  |  |  |  |  |  |  |
| High |  |  |  | Ref. |  | Ref. |  | Ref. | Ref. |
| Mid |  |  |  | 1.15 (0.66-1.99) |  | 1.10 (0.63-1.91) |  | 1.01 (0.58-1.78) | 1.12 (0.63-2.01) |
| Low |  |  |  | 1.16 (0.53-2.54) |  | 0.95 (0.42-2.13) |  | 0.85 (0.37-1.94) | 0.70 (0.29-1.67) |
| Marital status |  |  |  |  |  |  |  |  |  |
| Married/cohabiting |  |  |  | Ref. |  | Ref. |  | Ref. | Ref. |
| Never married |  |  |  | 2.04 (0.97-4.33) |  | 2.17 (1.02-4.62)* |  | 1.77 (0.82-3.80) | 1.94 (0.86-4.39) |
| Divorced/widowed |  |  |  | 5.05 (3.10-8.23)*** |  | 5.15 (3.15-8.42)*** |  | 4.56 (2.76-7.52)*** | 5.11 (3.02-8.65)*** |
| Household finances |  |  |  |  |  |  |  |  |  |
| Good/very good |  |  |  | Ref. |  | Ref. |  | Ref. | Ref. |
| Average |  |  |  | 3.07 (1.29-7.29)* |  | 3.01 (1.25-7.22)* |  | 3.50 (1.41-8.68)** | 3.48 (1.34-9.09)* |
| Bad/very bad |  |  |  | 9.14 (3.56-23.48)*** |  | 8.00 (3.08-20.81)*** |  | 7.53 (2.82-20.14)*** | 6.92 (2.46-19.46)*** |
| Location (Rural) |  |  |  | 0.87 (0.54-1.41) |  | 0.94 (0.58-1.52) |  | 0.88 (0.53-1.44) | 1.00 (0.60-1.66) |
| Self-rated health |  |  |  |  |  |  |  |  |  |
| Good/very good |  |  |  |  |  | Ref. |  | Ref. | Ref. |
| Fair |  |  |  |  |  | 1.11 (0.61-2.02) |  | 1.02 (0.55-1.89) | 0.85 (0.45-1.60) |
| Poor/very poor |  |  |  |  |  | 2.41 (1.19-4.87)* |  | 2.25 (1.10-4.60)* | 0.96 (0.44-2.10) |
| Social support |  |  |  |  |  |  |  |  |  |
| High |  |  |  |  |  |  |  | Ref. | Ref. |
| Medium |  |  |  |  |  |  |  | 3.05 (1.61-5.77)** | 2.36 (1.22-4.58)* |
| Low |  |  |  |  |  |  |  | 7.68 (3.46-17.03)*** | 6.67 (2.85-15.62)*** |
| Psychological distress |  |  |  |  |  |  |  |  | 1.33 (1.23-1.44)*** |
| Pseudo *R^2^* (Nagelkerke) |  | .04 |  | .26 |  | .27 |  | .31 | .37 |

OR: Odds ratio; CI: Confidence interval; Ref: Reference category; * p<.05, ** p<.01, *** p<.001.

**Table 4.** Food insecurity and loneliness in Georgia (*n* = 2006)

|  |  | **Model 1** |  | **Model 2** |  | **Model 3** |  | **Model 4** | **Model 5** |
| --- | --- | --- | --- | --- | --- | --- | --- | --- | --- |
|  |  | OR (95%CI) |  | OR (95%CI) |  | OR (95%CI) |  | OR (95%CI) | OR (95%CI) |
| Food insecurity |  |  |  |  |  |  |  |  |  |
| None |  | Ref. |  | Ref. |  | Ref. |  | Ref. | Ref. |
| Moderate |  | 2.06 (1.43-2.96)*** |  | 1.26 (0.83-1.91) |  | 1.18 (0.78-1.80) |  | 1.18 (0.77-1.80) | 1.10 (0.71-1.70) |
| Severe |  | 4.54 (3.05-6.76)*** |  | 1.68 (1.02-2.76)* |  | 1.54 (0.93-2.55) |  | 1.42 (0.85-2.38) | 1.27 (0.74-2.16) |
| Sex (Female) |  |  |  | 1.71 (1.18-2.48)** |  | 1.55 (1.07-2.26)* |  | 1.64 (1.11-2.42)* | 1.43 (0.96-2.13) |
| Age |  |  |  |  |  |  |  |  |  |
| 18-34 |  |  |  | Ref. |  | Ref. |  | Ref. | Ref. |
| 35-59 |  |  |  | 2.38 (1.46-3.87)** |  | 1.86 (1.12-3.10)* |  | 1.73 (1.04-2.89)* | 1.69 (1.00-2.86)* |
| ≥60 |  |  |  | 2.63 (1.54-4.47)*** |  | 1.80 (1.03-3.16)* |  | 1.60 (0.90-2.84) | 1.60 (0.89-2.88) |
| Education |  |  |  |  |  |  |  |  |  |
| High |  |  |  | Ref. |  | Ref. |  | Ref. | Ref. |
| Mid |  |  |  | 1.00 (0.70-1.42) |  | 0.93 (0.65-1.33) |  | 0.85 (0.59-1.22) | 0.82 (0.56-1.19) |
| Low |  |  |  | 1.06 (0.60-1.90) |  | 0.96 (0.53-1.72) |  | 0.87 (0.47-1.60) | 0.74 (0.39-1.40) |
| Marital status |  |  |  |  |  |  |  |  |  |
| Married/cohabiting |  |  |  | Ref. |  | Ref. |  | Ref. | Ref. |
| Never married |  |  |  | 3.89 (2.45-6.18)*** |  | 4.17 (2.61-6.68)*** |  | 3.80 (2.35-6.16)*** | 3.90 (2.38-6.37)*** |
| Divorced/widowed |  |  |  | 7.10 (4.87-10.35)*** |  | 6.98 (4.78 -10.20)*** |  | 6.72 (4.55-9.91)*** | 6.70 (4.50-9.97)*** |
| Household finances |  |  |  |  |  |  |  |  |  |
| Good/very good |  |  |  | Ref. |  | Ref. |  | Ref. | Ref. |
| Average |  |  |  | 1.94 (0.58-6.49) |  | 1.88 (0.56-6.32) |  | 1.81 (0.54-6.07) | 1.78 (0.51-6.23) |
| Bad/very bad |  |  |  | 4.20 (1.24-14.19)* |  | 3.40 (1.00-11.59) |  | 3.23 (0.95-11.03) | 2.75 (0.77-9.78) |
| Location (Rural) |  |  |  | 0.97 (0.71-1.33) |  | 0.93 (0.67-1.28) |  | 0.88 (0.63-1.22) | 0.86 (0.62-1.21) |
| Self-rated health |  |  |  |  |  |  |  |  |  |
| Good/very good |  |  |  |  |  | Ref. |  | Ref. | Ref. |
| Fair |  |  |  |  |  | 1.66 (0.94-2.92) |  | 1.67 (0.94-2.99) | 1.33 (0.74-2.41) |
| Poor/very poor |  |  |  |  |  | 2.89 (1.60-5.21)*** |  | 3.00 (1.64-5.48)*** | 1.35 (0.70-2.61) |
| Social support |  |  |  |  |  |  |  |  |  |
| High |  |  |  |  |  |  |  | Ref. | Ref. |
| Medium |  |  |  |  |  |  |  | 1.42 (0.67-3.01) | 1.21 (0.57-2.57) |
| Low |  |  |  |  |  |  |  | 27.83 (9.13-84.82)*** | 20.81 (6.71-64.55)*** |
| Psychological distress |  |  |  |  |  |  |  |  | 1.24 (1.17-1.32)*** |
| Pseudo *R^2^* (Nagelkerke) |  | .06 |  | .26 |  | .28 |  | .32 | .36 |

OR: Odds ratio; CI: Confidence interval; Ref: Reference category; * p<.05, ** p<.01, *** p<.001.

**Table 5.** Food insecurity and loneliness in Kazakhstan (*n* = 1616)

|  |  | **Model 1** |  | **Model 2** |  | **Model 3** |  | **Model 4** | **Model 5** |
| --- | --- | --- | --- | --- | --- | --- | --- | --- | --- |
|  |  | OR (95%CI) |  | OR (95%CI) |  | OR (95%CI) |  | OR (95%CI) | OR (95%CI) |
| Food insecurity |  |  |  |  |  |  |  |  |  |
| None |  | Ref. |  | Ref. |  | Ref. |  | Ref. | Ref. |
| Moderate |  | 4.65 (2.87-7.52)*** |  | 3.26 (1.92-5.55)*** |  | 3.18 (1.87-5.41)*** |  | 2.81 (1.63-4.87)*** | 2.38 (1.35-4.19)** |
| Severe |  | 9.99 (4.73-21.11)*** |  | 5.34 (2.21-12.88)*** |  | 5.11 (2.11-12.35)*** |  | 4.13 (1.64-10.39)** | 3.84 (1.48-9.98)** |
| Sex (Female) |  |  |  | 1.42 (0.85-2.40) |  | 1.39 (0.82-2.35) |  | 1.41 (0.83-2.40) | 1.21 (0.71-2.08) |
| Age |  |  |  |  |  |  |  |  |  |
| 18-34 |  |  |  | Ref. |  | Ref. |  | Ref. | Ref. |
| 35-59 |  |  |  | 2.23 (1.07-4.62)* |  | 2.08 (0.99-4.34) |  | 1.82 (0.87-3.79) | 1.83 (0.87-3.87) |
| ≥60 |  |  |  | 2.61 (1.11-6.14)* |  | 2.20 (0.90-5.37) |  | 2.09 (0.86-5.05) | 1.99 (0.80-4.94) |
| Education |  |  |  |  |  |  |  |  |  |
| High |  |  |  | Ref. |  | Ref. |  | Ref. | Ref. |
| Mid |  |  |  | 1.36 (0.73-2.51) |  | 1.29 (0.70-2.40) |  | 1.30 (0.69-2.43) | 1.42 (0.74-2.72) |
| Low |  |  |  | 1.41 (0.62-3.23) |  | 1.34 (0.59-3.09) |  | 1.37 (0.59-3.17) | 1.37 (0.57-3.29) |
| Marital status |  |  |  |  |  |  |  |  |  |
| Married/cohabiting |  |  |  | Ref. |  | Ref. |  | Ref. | Ref. |
| Never married |  |  |  | 2.51 (1.11-5.67)* |  | 2.64 (1.16-6.02)* |  | 2.30 (1.01-5.22)* | 2.60 (1.13-5.99)* |
| Divorced/widowed |  |  |  | 5.84 (3.30-10.35)*** |  | 5.81 (3.27-10.30)*** |  | 5.34 (2.98-9.56)*** | 5.08 (2.81-9.21)*** |
| Household finances |  |  |  |  |  |  |  |  |  |
| Good/very good |  |  |  | Ref. |  | Ref. |  | Ref. | Ref. |
| Average |  |  |  | 1.25 (0.65-2.43) |  | 1.18 (0.61-2.31) |  | 1.12 (0.57-2.19) | 1.04 (0.53-2.06) |
| Bad/very bad |  |  |  | 2.59 (1.12-6.00)* |  | 2.27 (0.96-5.37) |  | 1.84 (0.76-4.47) | 1.54 (0.62-3.78) |
| Location (Rural) |  |  |  | 0.80 (0.48-1.33) |  | 0.80 (0.48-1.33) |  | 0.75 (0.45-1.27) | 0.71 (0.42-1.20) |
| Self-rated health |  |  |  |  |  |  |  |  |  |
| Good/very good |  |  |  |  |  | Ref. |  | Ref. | Ref. |
| Fair |  |  |  |  |  | 1.47 (0.77-2.79) |  | 1.45 (0.76-2.76) | 1.13 (0.58-2.21) |
| Poor/very poor |  |  |  |  |  | 1.74 (0.74-4.07) |  | 1.64 (0.70-3.85) | 0.91 (0.37-2.25) |
| Social support |  |  |  |  |  |  |  |  |  |
| High |  |  |  |  |  |  |  | Ref. | Ref. |
| Medium |  |  |  |  |  |  |  | 1.95 (0.96-3.98) | 1.70 (0.82-3.53) |
| Low |  |  |  |  |  |  |  | 3.92 (1.48-10.39)** | 3.05 (1.13-8.24)* |
| Psychological distress |  |  |  |  |  |  |  |  | 1.27 (1.16-1.39)*** |
| Pseudo *R^2^* (Nagelkerke) |  | .10 |  | .25 |  | .25 |  | .26 | .30 |

OR: Odds ratio; CI: Confidence interval; Ref: Reference category; * p<.05, ** p<.01, *** p<.001.

**Table 6.** Food insecurity and loneliness in Kyrgyzstan (*n* = 1719)

|  |  | **Model 1** |  | **Model 2** |  | **Model 3** |  | **Model 4** | **Model 5** |
| --- | --- | --- | --- | --- | --- | --- | --- | --- | --- |
|  |  | OR (95%CI) |  | OR (95%CI) |  | OR (95%CI) |  | OR (95%CI) | OR (95%CI) |
| Food insecurity |  |  |  |  |  |  |  |  |  |
| None |  | Ref. |  | Ref. |  | Ref. |  | Ref. | Ref. |
| Moderate |  | 1.71 (1.12-2.60)* |  | 1.25 (0.80-1.97) |  | 1.27 (0.80-2.00) |  | 1.29 (0.81-2.06) | 1.27 (0.79-2.04) |
| Severe |  | 3.43 (2.17-5.41)*** |  | 2.03 (1.20-3.44)** |  | 1.98 (1.16-3.37)* |  | 2.16 (1.25-3.71)** | 2.12 (1.23-3.66)** |
| Sex (Female) |  |  |  | 0.89 (0.58-1.35) |  | 0.82 (0.54-1.26) |  | 0.89 (0.57-1.37) | 0.81 (0.52-1.26) |
| Age |  |  |  |  |  |  |  |  |  |
| 18-34 |  |  |  | Ref. |  | Ref. |  | Ref. | Ref. |
| 35-59 |  |  |  | 0.94 (0.57-1.55) |  | 0.79 (0.47-1.33) |  | 0.73 (0.43-1.25) | 0.70 (0.41-1.20) |
| ≥60 |  |  |  | 1.53 (0.83-2.83) |  | 1.12 (0.58-2.14) |  | 1.12 (0.57-2.18) | 1.02 (0.52-2.01) |
| Education |  |  |  |  |  |  |  |  |  |
| High |  |  |  | Ref. |  | Ref. |  | Ref. | Ref. |
| Mid |  |  |  | 1.27 (0.74-2.18) |  | 1.22 (0.71-2.08) |  | 1.27 (0.73-2.22) | 1.27 (0.72-2.22) |
| Low |  |  |  | 1.16 (0.63-2.16) |  | 1.12 (0.60-2.10) |  | 0.97 (0.51-1.86) | 0.92 (0.48-1.77) |
| Marital status |  |  |  |  |  |  |  |  |  |
| Married/cohabiting |  |  |  | Ref. |  | Ref. |  | Ref. | Ref. |
| Never married |  |  |  | 1.45 (0.79-2.67) |  | 1.56 (0.85-2.87) |  | 1.59 (0.86-2.95) | 1.73 (0.93-3.22) |
| Divorced/widowed |  |  |  | 7.13 (4.40-11.56)*** |  | 6.66 (4.09-10.84)*** |  | 5.42 (3.26-9.01)*** | 5.44 (3.25-9.11)*** |
| Household finances |  |  |  |  |  |  |  |  |  |
| Good/very good |  |  |  | Ref. |  | Ref. |  | Ref. | Ref. |
| Average |  |  |  | 1.63 (0.98-2.70) |  | 1.49 (0.89-2.48) |  | 1.42 (0.85-2.38) | 1.45 (0.86-2.45) |
| Bad/very bad |  |  |  | 3.80 (2.05-7.05)*** |  | 2.98 (1.57-5.66)** |  | 2.30 (1.19-4.47)* | 2.16 (1.11-4.22)* |
| Location (Rural) |  |  |  | 1.32 (0.88-1.97) |  | 1.34 (0.89-2.00) |  | 1.38 (0.91-2.10) | 1.37 (0.90-2.09) |
| Self-rated health |  |  |  |  |  |  |  |  |  |
| Good/very good |  |  |  |  |  | Ref. |  | Ref. | Ref. |
| Fair |  |  |  |  |  | 1.67 (1.01-2.75)* |  | 1.86 (1.12-3.10)* | 1.57 (0.93-2.64) |
| Poor/very poor |  |  |  |  |  | 2.53 (1.38-4.66)** |  | 2.83 (1.51-5.30)** | 1.82 (0.93-3.56) |
| Social support |  |  |  |  |  |  |  |  |  |
| High |  |  |  |  |  |  |  | Ref. | Ref. |
| Medium |  |  |  |  |  |  |  | 3.31 (1.88-5.83)*** | 3.15 (1.77-5.59)*** |
| Low |  |  |  |  |  |  |  | 7.21 (3.11-16.70)*** | 6.46 (2.71-15.43)*** |
| Psychological distress |  |  |  |  |  |  |  |  | 1.18 (1.09-1.28)*** |
| Pseudo *R^2^* (Nagelkerke) |  | .04 |  | .21 |  | .22 |  | .26 | .28 |

OR: Odds ratio; CI: Confidence interval; Ref: Reference category; * p<.05, ** p<.01, *** p<.001.

**Table 7.** Food insecurity and loneliness in Moldova (*n* = 1560)

|  |  | **Model 1** |  | **Model 2** |  | **Model 3** |  | **Model 4** | **Model 5** |
| --- | --- | --- | --- | --- | --- | --- | --- | --- | --- |
|  |  | OR (95%CI) |  | OR (95%CI) |  | OR (95%CI) |  | OR (95%CI) | OR (95%CI) |
| Food insecurity |  |  |  |  |  |  |  |  |  |
| None |  | Ref. |  | Ref. |  | Ref. |  | Ref. | Ref. |
| Moderate |  | 1.74 (1.31-2.31)*** |  | 1.19 (0.86-1.66) |  | 1.17 (0.84-1.64) |  | 1.10 (0.78-1.56) | 1.07 (0.75-1.52) |
| Severe |  | 3.38 (1.93-5.93)*** |  | 1.61 (0.82-3.14) |  | 1.51 (0.76-2.98) |  | 1.37 (0.66-2.82) | 1.28 (0.62-2.64) |
| Sex (Female) |  |  |  | 1.25 (0.92-1.70) |  | 1.22 (0.89-1.66) |  | 1.30 (0.94-1.80) | 1.18 (0.85-1.65) |
| Age |  |  |  |  |  |  |  |  |  |
| 18-34 |  |  |  | Ref. |  | Ref. |  | Ref. | Ref. |
| 35-59 |  |  |  | 0.90 (0.59-1.36) |  | 0.85 (0.55-1.31) |  | 0.82 (0.52-1.28) | 0.77 (0.49-1.22) |
| ≥60 |  |  |  | 1.26 (0.79-2.03) |  | 1.14 (0.69-1.90) |  | 1.07 (0.63-1.82) | 1.02 (0.60-1.76) |
| Education |  |  |  |  |  |  |  |  |  |
| High |  |  |  | Ref. |  | Ref. |  | Ref. | Ref. |
| Mid |  |  |  | 1.35 (0.89-2.05) |  | 1.35 (0.89-2.04) |  | 1.36 (0.89-2.09) | 1.32 (0.85-2.03) |
| Low |  |  |  | 1.32 (0.83-2.09) |  | 1.29 (0.81-2.05) |  | 1.10 (0.68-1.79) | 1.00 (0.61-1.63) |
| Marital status |  |  |  |  |  |  |  |  |  |
| Married/cohabiting |  |  |  | Ref. |  | Ref. |  | Ref. | Ref. |
| Never married |  |  |  | 0.91 (0.53-1.56) |  | 0.91 (0.53-1.57) |  | 0.74 (0.42-1.31) | 0.71 (0.40-1.25) |
| Divorced/widowed |  |  |  | 5.36 (3.82-7.53)*** |  | 5.31 (3.78-7.46)*** |  | 4.42 (3.09-6.31)*** | 4.64 (3.23-6.67)*** |
| Household finances |  |  |  |  |  |  |  |  |  |
| Good/very good |  |  |  | Ref. |  | Ref. |  | Ref. | Ref. |
| Average |  |  |  | 1.21 (0.81-1.79) |  | 1.18 (0.79-1.76) |  | 1.13 (0.75-1.71) | 1.07 (0.71-1.63) |
| Bad/very bad |  |  |  | 1.54 (0.97-2.45) |  | 1.46 (0.91-2.35) |  | 1.12 (0.68-1.84) | 0.95 (0.57-1.58) |
| Location (Rural) |  |  |  | 1.61 (1.16-2.25)** |  | 1.61 (1.15-2.24)** |  | 1.66 (1.17-2.34)** | 1.69 (1.19-2.39)** |
| Self-rated health |  |  |  |  |  |  |  |  |  |
| Good/very good |  |  |  |  |  | Ref. |  | Ref. | Ref. |
| Fair |  |  |  |  |  | 1.08 (0.74-1.58) |  | 1.15 (0.77-1.71) | 1.02 (0.68-1.53) |
| Poor/very poor |  |  |  |  |  | 1.31 (0.83-2.05) |  | 1.25 (0.78-2.00) | 0.86 (0.52-1.41) |
| Social support |  |  |  |  |  |  |  |  |  |
| High |  |  |  |  |  |  |  | Ref. | Ref. |
| Medium |  |  |  |  |  |  |  | 1.83 (1.09-3.07)* | 1.58 (0.93-2.68) |
| Low |  |  |  |  |  |  |  | 7.21 (4.64-11.18)*** | 6.53 (4.17-10.21)*** |
| Psychological distress |  |  |  |  |  |  |  |  | 1.17 (1.11-1.23)*** |
| Pseudo *R^2^* (Nagelkerke) |  | .03 |  | .21 |  | .21 |  | .28 | 31 |

OR: Odds ratio; CI: Confidence interval; Ref: Reference category; * p<.05, ** p<.01, *** p<.001.

**Table 8.** Food insecurity and loneliness in Russia (*n* = 2336)

|  |  | **Model 1** |  | **Model 2** |  | **Model 3** |  | **Model 4** | **Model 5** |
| --- | --- | --- | --- | --- | --- | --- | --- | --- | --- |
|  |  | OR (95%CI) |  | OR (95%CI) |  | OR (95%CI) |  | OR (95%CI) | OR (95%CI) |
| Food insecurity |  |  |  |  |  |  |  |  |  |
| None |  | Ref. |  | Ref. |  | Ref. |  | Ref. | Ref. |
| Moderate |  | 3.60 (2.56-5.08)*** |  | 2.26 (1.54-3.33)*** |  | 2.30 (1.56-3.38)*** |  | 2.15 (1.44-3.19)*** | 2.00 (1.33-2.99)** |
| Severe |  | 8.25 (5.01-13.57)*** |  | 4.36 (2.41-7.89)*** |  | 3.91 (2.14-7.15)*** |  | 3.62 (1.96-6.70)*** | 3.60 (1.90-6.82)*** |
| Sex (Female) |  |  |  | 1.52 (1.02-2.27)* |  | 1.43 (0.95-2.15) |  | 1.56 (1.03-2.37)* | 1.44 (0.94-2.22) |
| Age |  |  |  |  |  |  |  |  |  |
| 18-34 |  |  |  | Ref. |  | Ref. |  | Ref. | Ref. |
| 35-59 |  |  |  | 0.75 (0.43-1.33) |  | 0.61 (0.33-1.10) |  | 0.60 (0.33-1.09) | 0.55 (0.30-1.03) |
| ≥60 |  |  |  | 1.75 (0.96-3.17) |  | 1.11 (0.57-2.15) |  | 1.10 (0.57-2.15) | 1.07 (0.54-2.10) |
| Education |  |  |  |  |  |  |  |  |  |
| High |  |  |  | Ref. |  | Ref. |  | Ref. | Ref. |
| Mid |  |  |  | 1.33 (0.77-2.29) |  | 1.26 (0.73-2.19) |  | 1.23 (0.71-2.15) | 1.14 (0.64-2.00) |
| Low |  |  |  | 2.38 (1.24-4.54)** |  | 2.00 (1.03-3.88)* |  | 1.74 (0.89-3.41) | 1.65 (0.83-3.29) |
| Marital status |  |  |  |  |  |  |  |  |  |
| Married/cohabiting |  |  |  | Ref. |  | Ref. |  | Ref. | Ref. |
| Never married |  |  |  | 2.13 (1.12-4.04)* |  | 2.13 (1.12-4.06)* |  | 1.96 (1.03-3.74)* | 1.79 (0.92-3.51) |
| Divorced/widowed |  |  |  | 4.05 (2.69-6.10)*** |  | 4.08 (2.70-6.18)*** |  | 3.63 (2.38-5.54)*** | 3.80 (2.47-5.84)*** |
| Household finances |  |  |  |  |  |  |  |  |  |
| Good/very good |  |  |  | Ref. |  | Ref. |  | Ref. | Ref. |
| Average |  |  |  | 2.53 (1.14-5.62)* |  | 2.35 (1.05-5.27)* |  | 2.26 (1.01-5.07)* | 2.20 (0.97-4.97) |
| Bad/very bad |  |  |  | 4.18 (1.78-9.82)** |  | 3.31 (1.39-7.88)** |  | 2.99 (1.25-7.15)* | 2.75 (1.14-6.66)* |
| Location (Rural) |  |  |  | 1.34 (0.93-1.94) |  | 1.30 (0.90-1.89) |  | 1.23 (0.84-1.79) | 1.26 (0.85-1.85) |
| Self-rated health |  |  |  |  |  |  |  |  |  |
| Good/very good |  |  |  |  |  | Ref. |  | Ref. | Ref. |
| Fair |  |  |  |  |  | 1.29 (0.75-2.24) |  | 1.26 (0.72-2.19) | 0.89 (0.50-1.58) |
| Poor/very poor |  |  |  |  |  | 2.94 (1.56-5.58)** |  | 2.83 (1.49-5.39)** | 1.22 (0.61-2.46) |
| Social support |  |  |  |  |  |  |  |  |  |
| High |  |  |  |  |  |  |  | Ref. | Ref. |
| Medium |  |  |  |  |  |  |  | 2.06 (1.10-3.87)* | 1.83 (0.95-3.52) |
| Low |  |  |  |  |  |  |  | 3.13 (1.69-5.79)*** | 2.90 (1.53-5.49)** |
| Psychological distress |  |  |  |  |  |  |  |  | 1.25 (1.18-1.33)*** |
| Pseudo *R^2^* (Nagelkerke) |  | .09 |  | .27 |  | .28 |  | .30 | .35 |

OR: Odds ratio; CI: Confidence interval; Ref: Reference category; * p<.05, ** p<.01, *** p<.001.

**Table 9.** Food insecurity and loneliness in Ukraine (*n* = 1634)

|  |  | **Model 1** |  | **Model 2** |  | **Model 3** |  | **Model 4** | **Model 5** |
| --- | --- | --- | --- | --- | --- | --- | --- | --- | --- |
|  |  | OR (95%CI) |  | OR (95%CI) |  | OR (95%CI) |  | OR (95%CI) | OR (95%CI) |
| Food insecurity |  |  |  |  |  |  |  |  |  |
| None |  | Ref. |  | Ref. |  | Ref. |  | Ref. | Ref. |
| Moderate |  | 1.81 (1.23-2.68)** |  | 1.21 (0.78-1.87) |  | 1.19 (0.77-1.86) |  | 1.20 (0.76-1.88) | 1.33 (0.84-2.12) |
| Severe |  | 5.92 (3.87-9.06)*** |  | 3.05 (1.80-5.19)*** |  | 2.84 (1.65-4.89)*** |  | 2.46 (1.40-4.31)** | 2.68 (1.50-4.78)** |
| Sex (Female) |  |  |  | 1.42 (0.92-2.19) |  | 1.29 (0.83-2.01) |  | 1.42 (0.90-2.23) | 1.14 (0.71-1.81) |
| Age |  |  |  |  |  |  |  |  |  |
| 18-34 |  |  |  | Ref. |  | Ref. |  | Ref. | Ref. |
| 35-59 |  |  |  | 2.00 (1.00-4.01) |  | 1.41 (0.68-2.95) |  | 1.27 (0.61-2.65) | 1.13 (0.54-2.38) |
| ≥60 |  |  |  | 3.85 (1.87-7.92)*** |  | 2.20 (1.01-4.78)* |  | 1.95 (0.89-4.27) | 1.90 (0.86-4.19) |
| Education |  |  |  |  |  |  |  |  |  |
| High |  |  |  | Ref. |  | Ref. |  | Ref. | Ref. |
| Mid |  |  |  | 1.29 (0.81-2.05) |  | 1.23 (0.76-1.97) |  | 1.20 (0.73-1.96) | 1.18 (0.71-1.96) |
| Low |  |  |  | 1.54 (0.85-2.80) |  | 1.35 (0.74-2.49) |  | 1.27 (0.68-2.38) | 1.26 (0.66-2.41) |
| Marital status |  |  |  |  |  |  |  |  |  |
| Married/cohabiting |  |  |  | Ref. |  | Ref. |  | Ref. | Ref. |
| Never married |  |  |  | 5.35 (2.59-11.06)*** |  | 5.82 (2.77-12.24)*** |  | 4.81 (2.28-10.18)*** | 4.23 (1.99-8.99)*** |
| Divorced/widowed |  |  |  | 5.41 (3.43-8.54)*** |  | 5.51 (3.48-8.74)*** |  | 4.44 (2.77-7.14)*** | 4.59 (2.82-7.46)*** |
| Household finances |  |  |  |  |  |  |  |  |  |
| Good/very good |  |  |  | Ref. |  | Ref. |  | Ref. | Ref. |
| Average |  |  |  | 1.58 (0.79-3.16) |  | 1.40 (0.69-2.84) |  | 1.59 (0.76-3.31) | 1.64 (0.76-3.56) |
| Bad/very bad |  |  |  | 2.04 (0.96-4.33) |  | 1.34 (0.61-2.94) |  | 1.32 (0.59-2.96) | 1.03 (0.44-2.42) |
| Location (Rural) |  |  |  | 1.23 (0.82-1.84) |  | 1.11 (0.73-1.67) |  | 1.06 (0.69-1.63) | 0.97 (0.63-1.51) |
| Self-rated health |  |  |  |  |  |  |  |  |  |
| Good/very good |  |  |  |  |  | Ref. |  | Ref. | Ref. |
| Fair |  |  |  |  |  | 2.09 (1.09-4.01)* |  | 2.13 (1.10-4.12)* | 1.64 (0.83-3.26) |
| Poor/very poor |  |  |  |  |  | 4.70 (2.27-9.72)*** |  | 4.73 (2.26-9.87)*** | 2.50 (1.14-5.47)* |
| Social support |  |  |  |  |  |  |  |  |  |
| High |  |  |  |  |  |  |  | Ref. | Ref. |
| Medium |  |  |  |  |  |  |  | 3.21 (1.63-6.33)** | 3.18 (1.61-6.31)** |
| Low |  |  |  |  |  |  |  | 6.33 (3.40-11.78)*** | 6.03 (3.16-11.49)*** |
| Psychological distress |  |  |  |  |  |  |  |  | 1.26 (1.17-1.35)*** |
| Pseudo *R^2^* (Nagelkerke) |  | .08 |  | .27 |  | .29 |  | .33 | .38 |

OR: Odds ratio; CI: Confidence interval; Ref: Reference category; * p<.05, ** p<.01, *** p<.001.
